# Long-term Performance and Durability of Biomaterials and Implant Designs in Total Joint Arthroplasty and Spinal Fusion

**DOI:** 10.26502/josm.511500256

**Published:** 2026-03-13

**Authors:** David Parvizi, Artin Allahverdian, Gregory Ayzenberg, Sugeeth Kandikattu, Ramtin Sahafi, Devendra K Agrawal

**Affiliations:** Department of Translational Research, College of Osteopathic Medicine of the Pacific, Western University of Health Sciences, Pomona CA 91766, USA

**Keywords:** Arthroplasty, Additive manufacturing, Biomaterials, Cobalt-chromium, Orthopedic implants, Osseointegration, Spinal fusion, Stress shielding, Surface modification, Titanium alloys, Total joint arthroplasty, Ultra-high–molecular-weight polyethylene (UHMWPE), Wear debris

## Abstract

Advancements in biomaterials and implant design has created a lasting impact on orthopedic surgery. Changes such as implant longevity, biocompatibility, and patient satisfaction have been attained with the use of new biomaterials. Contemporary implants have been able to balance mechanical strength, corrosion resistance, wear characteristics, and biological integration all while decreasing complications including infection and periprosthetic fractures. Metallic biomaterials include titanium alloys, cobalt-chromium alloys, and stainless steel. These materials excel in durability and have favorable strength-to-weight ratios. Although, there are challenges when introducing new biomaterials in orthopedic surgery. Stress shielding and wear-induced osteolysis and stress shielding. This has prompted improvements to implant geometry, surface modifications, and material compositions. Polymeric components such as ultra-high molecular weight polyethylene has undergone significant refinement over the last several years leading to a reduction in oxidative degradation and debris formation. Newer technologic advancements in porous coatings, bioactive surface treatments, additive manufacturing, and patient-specific implant design have shown to further enhance osseointegration and biomechanics compatibility. The interplay between material science, biomechanics, and host biology is essential for ideal implant optimization and reducing the number of revisions. Continued research integrating materials engineering and clinical outcomes will drive the next generation of durable, biologically integrated orthopedic implants.

## 1. Introduction

Degenerative joint and spinal diseases, primarily osteoarthritis, low back pain, and neck pain constitute a major global health burden. Prevalence and disability rates have been slowly rising each year due to aging populations and increased obesity. In 2021, musculoskeletal disorders affected 1.3 billion people worldwide, with osteoarthritis alone impacting 607 million people [1]. Prevalence of osteoarthritis is highest in high-income areas, with age-standardized rated surpassing 8,000 per 100,000 in high-income Asia Pacific and North America, and lowest in sub-Saharan Africa and Southeast Asia with rates around 5700 per 100,000 [2]. The knee is the most commonly affected joint and osteoarthritis and disability increases with age and are more common in women compared to men. The burden of degenerative joint disease is projected to rise further, with cases of osteoarthritis predicted to surpass 1.1 billion by 2050 [3]. Moreover, low back pain affects over 500 million people per year globally and is the top cause of disability in most countries and age groups. Over the last thirty years, the number of affected individuals and years lived with disability has increased significantly. The highest prevalence is found within working-age and older adults [4]. Degenerative joint disease and spinal diseases are among the most prevalent and most disabling conditions worldwide, with substantial impacts on the health system, economy, and quality of life.

The increasing demand for total joint arthroplasty and spinal fusion in the context of global burden and degenerative joint disease is driven by several factors. The most significant driver is the aging population, with a marked rise in the proportion of elderly individuals who are at high risk for osteoarthritis and degenerative spine conditions [5]. This demographic is projected to grow which will lead to a large increase in sheer procedure volume, most specifically in patients aged 65 and older for joint arthroplasty and spinal fusion [6]. Another factor includes the rising prevalence of degenerative joint disease. This rising incidence is due to an aging population, obesity, and sedentary lifestyles which results in more patients with advanced disease requiring surgery [7]. In the last decade, there has been a trend in offering surgical intervention to a wider patient population, which includes young and active individuals. Moreover, surgery has been offered to patients with less severe disease as well. This has been driven by improved surgical techniques, better implant longevity, and higher patient expectations for quality of life post-op [8]. As a result of these improved efficiencies, advances in perioperative management and anesthesia care has resulted in reduced complication rates which has expanded the eligible pool of surgical candidates. Increasing demand also comes from advancements in the health system and socioeconomic factors. Patients who now have expanded insurance coverage, higher education, and greater access to healthcare resources are associated with higher rates of spinal fusion and arthroplasty. However, regional variation exists based on surgeon enthusiasm [9]. The rising demand for joint arthroplasty and spinal fusion is multifactorial and takes into account demographic, economic, technological, and epidemiologic factors which all contribute to higher rates of arthroplasty.

The history and evolution of orthopedic implants in total joint arthroplasty and spinal fusion reflect major advances in biomaterials science, engineering, and surgical technique. Early joint arthroplasty in the 19th and 20th centuries were based on resection and interposition procedures. Once the introduction of general anesthesia was made and antiseptic techniques were integrated, more complex reconstructions were able to be performed. The creation of endoprosthetic replacements and the first modern total joint replacement in the 1970s signaled a turning point in the field. The use of metals including stainless steel, cobalt-chromium alloys, and titanium alloys became the standard for hip and knee arthroplasties [10]. In regards to spinal fusion, initial techniques used autografts and allografts, however, in the last several decades there has been a shift toward interbody devices made from metals such as titanium and polyetheretherketone (PEEK) [11]. Recent advancements have shifted toward ceramics, highly cross-linked polyethylene, nanotechnology, and 3D-printed personalized implants which all move in the direction of improved biocompatibility, mechanical stability, and long-term durability. The evolution of implant design and biomaterial choice all contribute to the need to reduce wear and complication rates surrounding arthroplasty and enhancing clinical outcomes [12].

Biomaterial selection and implant design are critical determinants of long-term outcomes for patients who undergo joint arthroplasty and spinal fusion, especially since the global burden of degenerative joint disease has been expanding. The decision of which biomaterial to use in each implant will determine implant longevity, osseointegration, wear particle generation, and the risk of complications such as loosening, infection, and periprosthetic osteolysis [13]. Combinations such as highly cross-linked polyethylene, ceramics, and advanced metal alloys have decreased wear and tear and improved the survivability of implants whereas a suboptimal choice of implant hardware can increase the rate of failure [14]. In spinal fusion, titanium and polyetheretherketone (PEEK) are amongst the two most common materials used for implants. Titanium offers high rates of osseointegration but can also cause stress shielding as a result of the high elastic modulus. PEEK is radiolucent and its modulus resembles bone, however it is bioinert and less osteoconductive [15]. Modifying the surface of implants have also shown to enhance bone ingrowth and implant stability. Modifications include increased roughness, porosity, and bioactive coatings, all of which reduce non-union and revision rates [16]. Moreover, design of the implant including geometry and tribological properties have shown to minimize micromotion, reducing wear, and distribution of load, all of which act to increase longevity of the joint implant. As demand for total joint arthroplasty increases, optimizing biomaterial selection and implant design is essential to improve patient outcomes and reduce healthcare burden [17].

The objective of this article is to critically evaluate and compare how various materials and design features can influence implant survival, complication rates, biological integration, and functional outcomes over time. This review highlights the strengths and limitations of commonly used biomaterials such as titanium, PEEK, ceramics, and polyethylene as well as compare design modifications including roughness, porosity, and tribological optimization. The goal is to inform clinical decision making and guide future innovation in implant technology.

## 2. Biomaterials in Orthopedic Implants

Titanium and its alloys, including titanium-6-aluminum-4vanadium (Ti-6Al-4V), are characterized by high mechanical strength, superior corrosion resistance, and excellent biocompatibility. In terms of mechanical strength, Ti-6Al-4V is substantially stronger compared to pure titanium. Its features include high tensile strength and fatigue resistance which makes it an exceptional choice for orthopedic and dental implants. It also includes an elastic modulus that is lower compared to stainless steel and cobalt-chromium alloys resulting in less stress shielding in bone applications [1]. Ti-6Al-4V is also able to form a stable and adherent oxide layer on its surface allowing resistance to corrosion when implanted. Treatments to the surface of the compound such as sandblasting, oxidation, and nanoconstruction improves the resistance to corrosion allowing for long-term implant stability [2]. Ti-6Al-4V is also widely recognized for its biocompatibility allowing for osseointegration and limited inflammatory response. However, one concern includes the release of vanadium and aluminum ions over time which can possibly lead to cytotoxic effects with high concentrations [3].

Cobalt-chromium alloys are an alternative to Ti-6Al-4V and have characteristic wear resistance and are used mainly in knee and hip prosthetics. The microstructure is made up of carbies and intermetallic compounds which improve surface hardness, providing significant protection against wear resistance. The fatigue properties of this compound is also favorable given its high fatigue strength and resistance to crack propagation, making it an essential choice for implant durability and longevity [4]. The manufacturing of this compound is done through laser surface melting which allows for improved fatigue and wear performance [5]. One issue found with cobalt-chromium ions is metal ion release. Although the compound is resistant to corrosion, wear and tribocorrosion can lead to the slow release of cobalt and chromium ions into adjacent tissues and even systemically. Cobalt has been shown to be associated with local cytotoxicity, inflammatory responses, and possible systemic cobalt toxicity, although this complication is exceedingly rare. Alloy composition, surface finish, manufacturing technique, and local pH are all contributors to the extent of cobalt release [6]. Techniques including surface coating and alloy modifications are currently under investigation in order to alleviate significant ion release [7].

Historically, stainless steel was used widely for orthopedic and dental implants due to its favorable mechanical properties, cost-effectiveness, and corrosion resistance [8]. Grade 316L stainless steel provided better corrosion resistance compared to previous stainless steel models as its carbon content was lower, as well as the presence of chromium which creates a passive oxide layer [9]. However, several factors have contributed to the declines in the use of stainless steel for permanent implants. The most significant factor is the susceptibility to localized corrosion in physiological environments. Corrosion is worsened due to the presence of chloride ions found in body fluids, further disrupting the passive oxide layer and speeding up corrosion. Corrosion results in the build up of metal ions such as nickel and chromium. These metals are associated with cytotoxicity, allergic reactions, and poor tissue response [10]. Long-term studies of previously used implants showed significant surface degradation and ion leaching, leading to failure of the implant and soft tissue reactions [11]. Advancements in biomaterials science has led to the use of titanium alloys and cobalt-chromium alloys as they offer superior corrosion resistance and improved biocompatibility [12].

**Figure 1:**
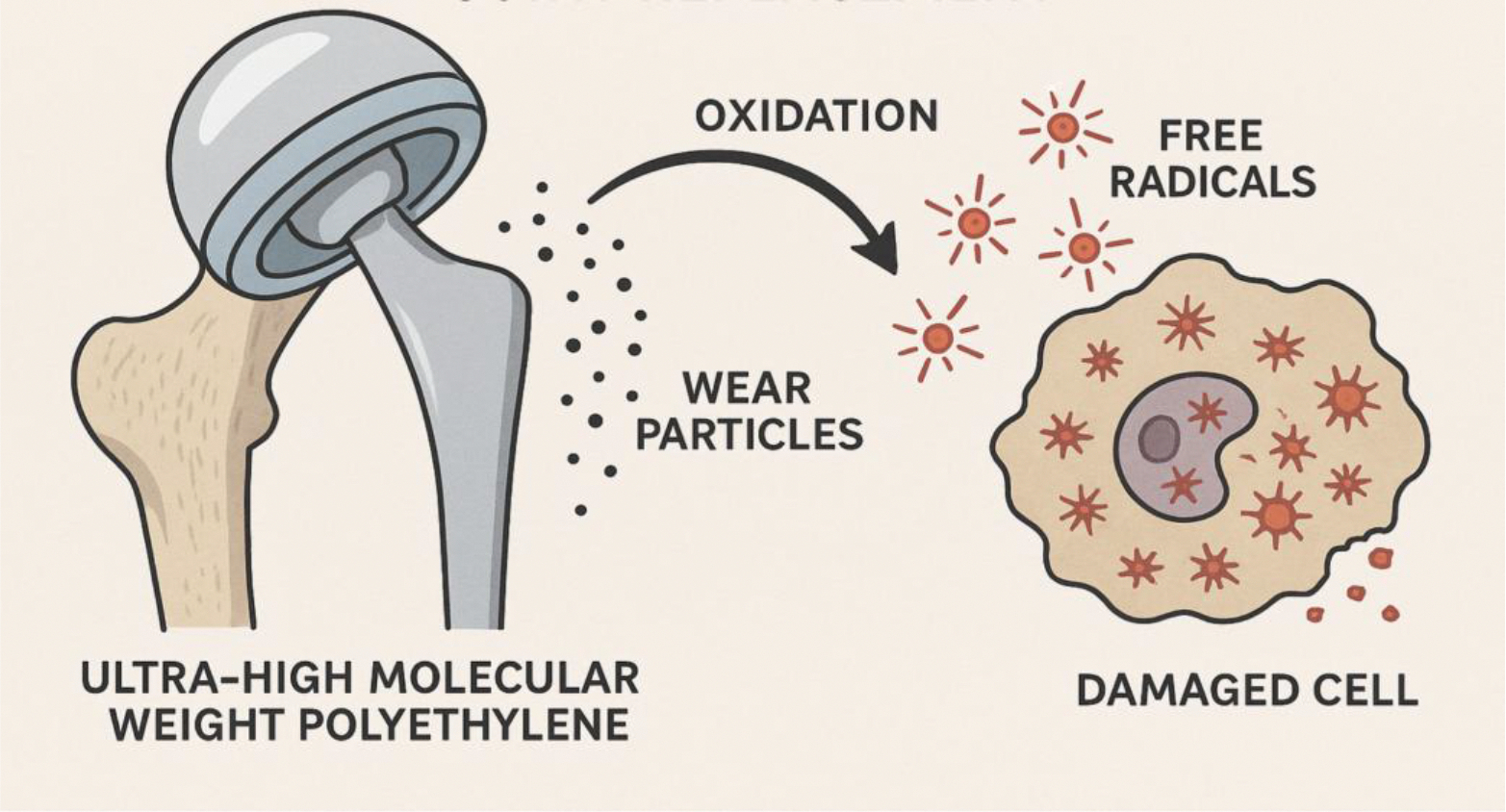
Ultra-high molecular weight polyethylene (UHMWPE) undergoes oxidative damage when free radicals from irradiation or in vivo exposure react with oxygen, forming oxidized species that weaken the polymer. Lipid absorption from synovial fluid, especially squalene, can further accelerate oxidation and compromise UHMWPE stability in joint arthroplasty.

Ultra-high molecular weight polyethylene (UHMWPE) has low wear rates, however, wear particle generation is a key contributor to osteolysis and implant loosening [13]. Highly crosslinked UHMWPE is made through irradiation and is associated with reduced wear rates in total hip arthroplasty leading to lower revision rates compared to traditional UHMWPE. However, studies have shown that in total knee arthroplasty, the benefits are not as significant due to differing kinematics and weight bearing [14]. The major concern for UHMWPE is oxidative degradation, particularly following irradiation which can create free radicals that reacts with oxygen and compromises the mechanical properties of the implant over time. Recent studies have shown that crosslinking innovations use vitamin E as an antioxidant, which can be blended or diffused into UHMWPE to scavenge free radicals and enhance the oxidative stability of the metal (Figure 1) [15].

Polyetheretherketone (PEEK) has shown to offer advantages in spinal fusion procedures due to its improved load sharing properties. The elastic modulus of PEEK is around 3.6–4.0 GPa which is very close to human bone (7–30 GPa) compared to a material such as titanium. This proximity to human bone reduces stress shielding and allows for more physiologic load transfer through the bone graft, leading to enhanced fusion and lower risk of implant subsidence and soft tissue degeneration [16]. Optimal load sharing with PEEK has been shown in several studies demonstrating increased stress within the bone graft and decreased endplate stress compared to titanium [17]. Although PEEK is a superior material, it does not come with any downsides. The concern is PEEK’s bioinertness center on its limited osseointegration and tendency to elicit a mild foreign body response. This compound is not able to naturally integrate with bone which can cause fibrous tissue formation at the bone-implant interface and can increase risk of non-union or delayed fusion [18]. Wear debris generation from PEEK is also a concern, as it can cause a local inflammatory response. Studies have shown that PEEK wear particles can be phagocytosed if the particle is between 0.1–10 micrometers which creates a biological response similar to that of UHMWPE. This reaction can cause mild macrophage infiltration and increased cytokine release leading to potential osteolysis (Figure 1). However, long-term studies are limited [19].

Alumina and zirconia implants are characterized by their high wear resistance, however, both materials are more brittle compared to metal alloys and can carry a risk of fracture. The most significant limitation to ceramic implants is their brittleness, which carries a higher risk for fracture, abnormal loading, trauma, and malpositioning. Alumina is more susceptible as it has low fracture roughness whereas zirconia can undergo phase transformation making it tougher and less susceptible [20]. Current literature suggests that fracture rates for modern alumina is approximately 0.02% and for alumina matrix composites the rate drops below 0.01% [21]. Adding zirconia to alumina and alumina to zirconia can increase fracture toughness and fatigue resistance which decreases rates of fractures and maintains good wear properties [22].

Hydroxyapatite coatings can enhance osseointegration in orthopedic implants through providing a bioactive, osteoconductive surface which is similar to the mineral phase of native bone. The similarity in composition of hydroxyapatite to mineral bone allows for rapid adsorption of proteins and growth factors, which enhances osteoblast adhesion, proliferation, and differentiation. Plasma spraying and electrochemical deposition spraying techniques optimizes the topography, crystallinity, and wettability of the hydroxyapatite coatings leading to improved cellular responses and earlier osteogenic activity [23]. The coating is also able to create a barrier for wear debris and corrosion, leading to long-term implant stability and reducing the risk of aseptic loosening. Overall, the effect creates a robust and durable bone-implant interface [24–40].

## 3. Implant Design Considerations

There are many factors that are put into consideration when choosing implants for joint arthroplasty and spinal fusion. The design, new innovations, and compatibility of the implants play a major role in the long-term success of patients. When looking at the design of implants, the surface finish and fixation methods play a vital role in decision making processes.

The surface finish is able to directly influence osseointegration, cellular response, stability and the rate of infection [41]. The surface roughness and porosity are able to enhance and promote the osteoblast attachment and proliferation which leads to better patient outcomes. Grit-blasting, acid-etching, plasma spraying and nano texturing accelerate bone ingrowth and integration. It is important to find the right balance of roughness when creating an implant, because excessively rough surfaces can actually impede cell proliferation and increase oxidative stress. Creating a surface finish with the right roughness and porousness improves vascularization, osteoblast attachment, fusion rates and implant loosening [42].

Other than the surface of the implants, fixation methods are also an important factor in implant considerations. There are two main methods: cemented and non-cemented methods. Cemented fixation is the gold standard, it utilizes polymethylmethacrylate bone cement to anchor the implant to the bone. These implants have shown great stability and long-term survival especially for older and osteoporotic patients [43]. On the other hand, non-cemented fixation relies on surrounding bone to grow into the porous surface of the implant. These implants are usually used in younger and more active patients [44].

**Figure 2:**
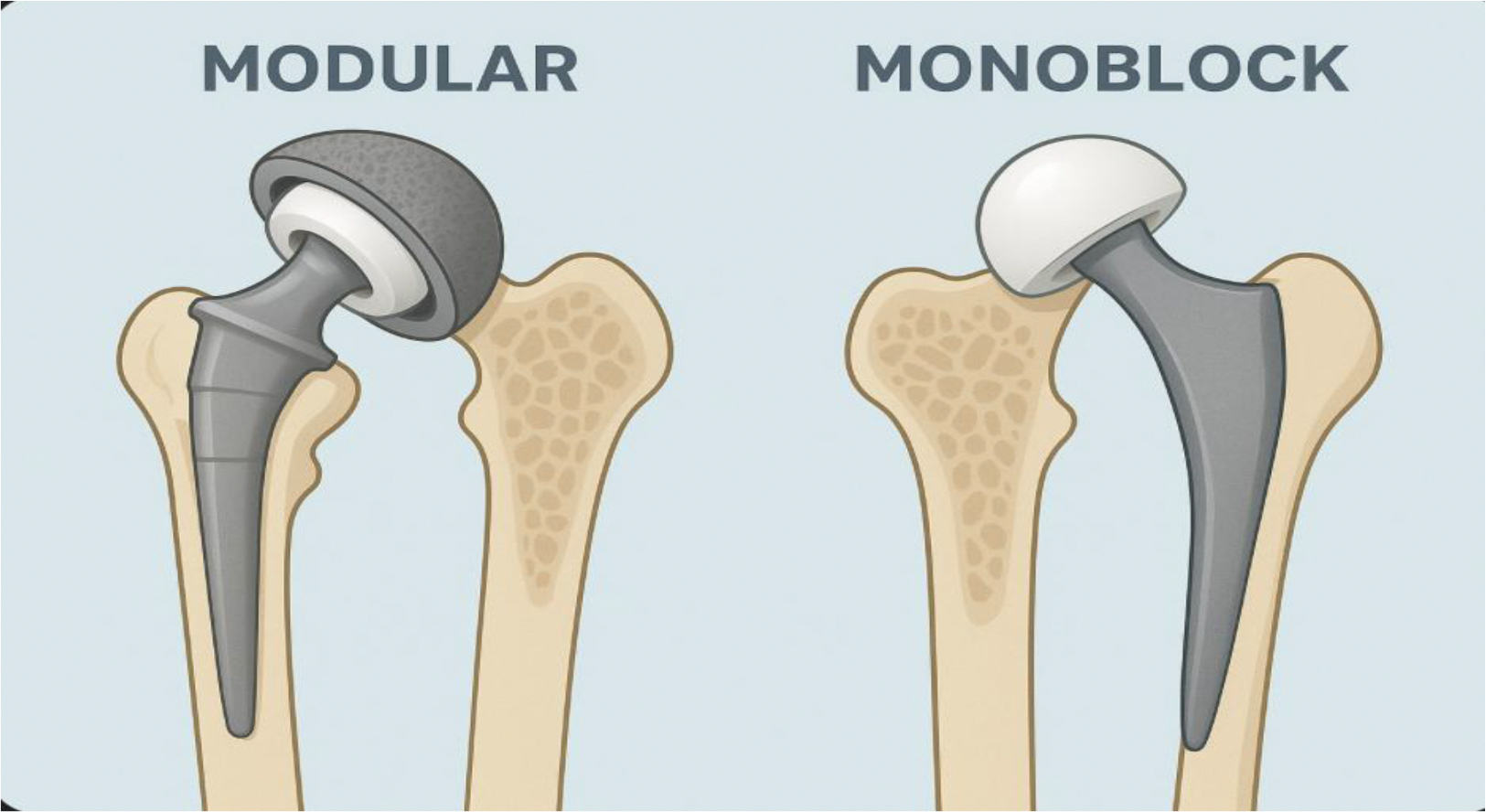
The difference between monoblock and modular designs for orthopedic implants is that monoblock implants are manufactured as a single, continuous piece, whereas modular implants are composed of multiple interlocking components that can be assembled intraoperatively to tailor fit, length, offset, and version to the patient’s anatomy.

Implants are generally made using one of two design configurations, the modular or monoblock designs, where each offers distinct structural and functional characteristics (Figure 2). The modular design offers flexibility in choosing the components size, alignment and fixation intraoperatively. Which allows for different parts to be exchanged without removing the entire implant. Compared to a monoblock design, the modular design has a higher rate of polyethylene wear and osteolysis [45]. The monoblock implants exhibit less early migration and loosening which lead to a better initial fixation and greater long-term durability. The monoblock designs pose challenges when changes need to be made. Since there are no replaceable parts, the whole implant will have to be taken out when a part of it is causing problems for the patients. These designs can lead to mechanical complications, including fretting, corrosion, and component dissociation [46].

There have been many new innovations in the creation of implants. Three-dimensional printing (3D) has shown to improve the quality of knee arthroplasty and spinal fusion. These new innovations have helped to increase the alignment, surgical precision, and overall clinical outcomes, especially in complex or uncommon cases. With detailed imaging from CT and MRI scans, 3D printer implants and surgical instruments are created to match the patient’s unique bone structures. In knee arthroplasty, these implants improve the outcomes in patients by achieving better alignment and reducing the soft tissue imbalances. With today’s technological advances, these 3D implants can be made within minutes [47]. In spinal fusion, 3D printing is used to create interbody cages, vertebral body replacements, and surgical guides. These specialized and personally tailored implants are especially useful in patients with deformities, tumors or revision surgeries [48]. Research has shown that using 3D printed implants can decrease operation times, reduce blood loss and improve fusion success rates. In addition, they can help surgeons refine their surgical techniques leading to less complications and surprises during the operation [49].

Load transfer and stress shielding are important determinants of the long-term success of the implants, as they play a vital role in bone remodeling, implant stability, and mechanical failure. Load transfer characterizes the manner in which the mechanical forces are transmitted from the implants to bone. This is why considering geometry, porosity, and surface consistency is integral to enable appropriate load transfer [50]. These designs help preserve the bones and aid in osseointegration. For example, when using partial knee arthroplasty where some original structures are preserved, the new implants lead to less stress shielding and better outcomes than with total knee arthroplasty [51].

Stress shielding emerges when an implant has a significantly higher stiffness than the surrounding bone which causes it to sustain different pressures. This can result in bone resorption, decreased bone mineral density, and alter the impact fixation [52].

In knee arthroplasty, this phenomenon is particularly evident when high density metallic components are used, leading to higher likelihood of periprosthetic bone loss. The stronger materials like cobalt-chromium might be substituted with titanium alloys or polyetheretherketone for better long-term results. Using highly porous and cementless designs also alleviates stress shielding by matching the mechanical properties of the bone and implant supporting bone maturation and growth [53].

## 4. Biomaterial Durability

### 4.1 Comparison of Different Bearing Surfaces

**Figure 3:**
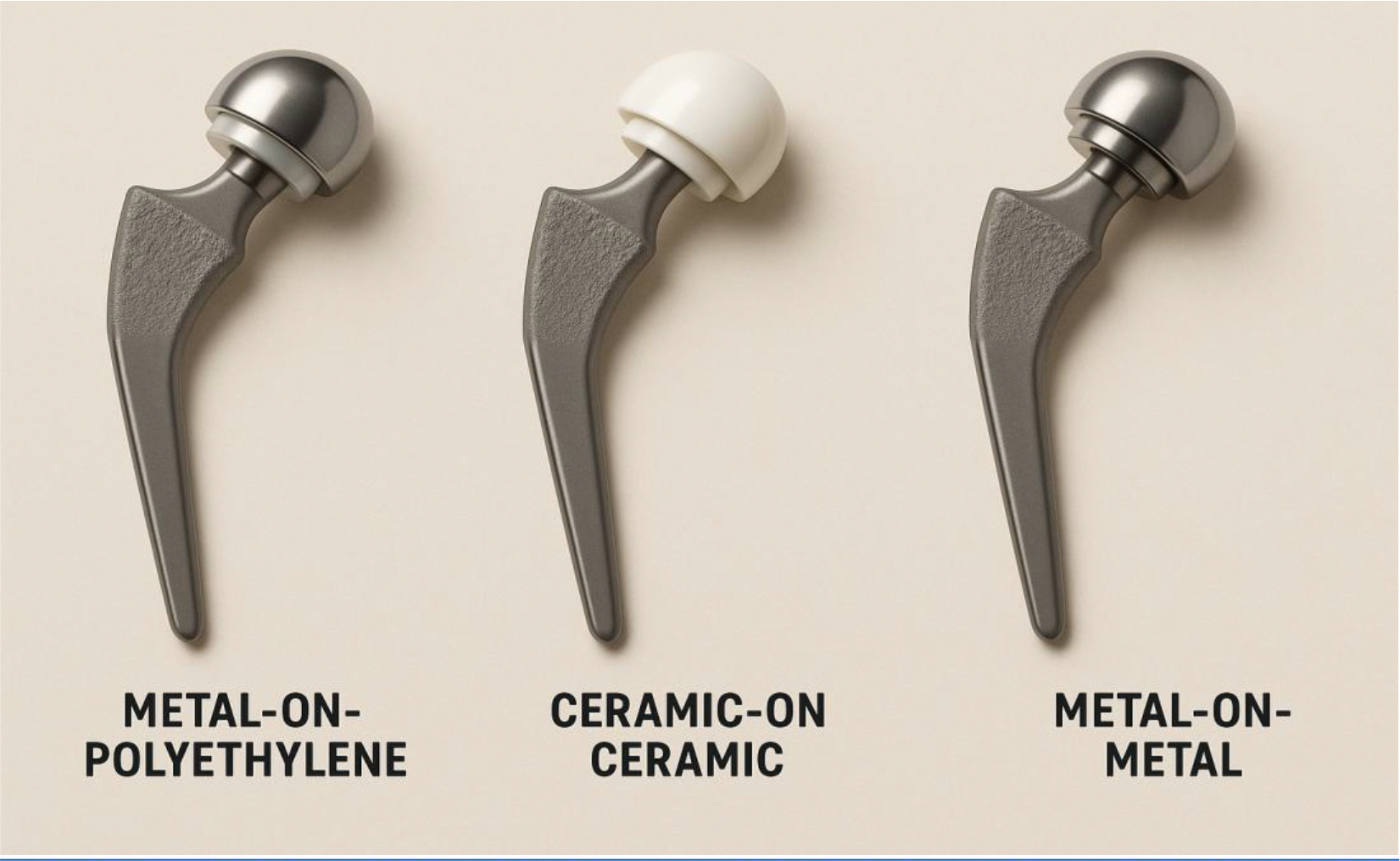
Different types of implants for the total hip arthroplasty.

The long-term performance of biomaterials in total hip arthroplasty (THA) is heavily influenced by the type of bearing surface used (Figure 3). Registry data from over 150,000 patients demonstrate that ceramic-on-ceramic (CoC) bearings have the highest 10-year implant survival (95.4%), followed closely by ceramic-on-highly cross-linked polyethylene (CoXLP, 94.3%) and metal-on-highly cross-linked polyethylene (MoXLP, 94.2%). In contrast, metal-on-metal (MoM) bearings show inferior survivorship (85.5%) and a higher risk of revision, particularly beyond five years [54–57]. These findings are corroborated by long-term cohort studies, which report 25-year survival rates exceeding 93% for third-generation CoC bearings and similar durability for CoXLP, with minimal osteolysis or component fracture [58].

Metal-on-polyethylene (MoP) bearings use highly cross-linked polyethylene (HXLPE), which allows them to demonstrate excellent long-term outcomes, with 10-year implant survival rates exceeding 94% and minimal wear-related revisions or osteolysis, even in younger, active patients [58–61]. Conventional polyethylene liners are associated with higher wear rates and increased risk of osteolysis and revision, but HXLPE has virtually eliminated these complications after 15–20 years of follow-up [62]. Ceramic-on-ceramic (CoC) bearings offer the lowest wear rates and near elimination of particle-induced osteolysis, with 10–25 year survivorship rates of 93–98% and minimal radiographic evidence of loosening or osteolysis [63–68]. However, CoC is associated with unique complications such as squeaking and rare ceramic fracture, though these events rarely require revision surgery [69–70]. Functional outcomes are comparable between HXLPE-based MoP and CoC bearings, as both are considered safe and durable options for most patients (Figure 3) [71].

Metal-on-metal (MoM) bearings, particularly with larger head sizes, have shown inferior long-term survivorship and higher complication rates, including adverse local tissue reactions, metallosis, and elevated serum metal ion levels [72–75]. Registry analyses consistently demonstrate increased risk of revision for MoM implants after two years, with the effect becoming more pronounced over time [76]. While small-head MoM designs may have better survival than large-head MoM, the risk of adverse reaction to metal debris and systemic metal ion exposure remains concerning, leading to a strong decline in their use [77]. Overall, HXLPE-based MoP and CoC bearings provide the most favorable long-term outcomes in total hip arthroplasty, while MoM bearings are associated with unacceptable risks and are no longer recommended for routine use [78–83].

### 4.2 Complications of Hardware in Total Hip Arthroplasty

There exists a variety of long-term complications leading to failure of total hip arthroplasty hardware. The complications that will be discussed include aseptic loosening, wear particle–induced osteolysis, and periprosthetic joint infection.

Aseptic loosening is the most frequent long-term cause of hardware failure in total joint arthroplasty, manifesting several years after implantation. This phenomenon results from chronic inflammation and bone resorption at the bone-implant interface, primarily driven by the host immune response to particulate wear debris from implant materials such as polyethylene, metal, or ceramic. This process involves activation of macrophages and osteoclasts, leading to progressive osteolysis and eventual loss of implant fixation, with revision rates for aseptic loosening remaining a significant burden despite advances in biomaterial design [84–89]. Wear particle–induced osteolysis is central to the pathogenesis of aseptic loosening. Micron- and nano-sized particles generated from bearing surfaces stimulate a local inflammatory response, characterized by cytokine release and the recruitment of osteoclasts, which resorb periprosthetic bone. The extent of osteolysis is influenced by patient activity, implant design, and material properties, with highly cross-linked polyethylene and advanced ceramics showing reduced particle generation and lower rates of osteolysis compared to conventional hardware [90–96]. However, once initiated, osteolysis is progressive and is currently only reversible by surgical intervention to remove the source of the wear [97].

Periprosthetic joint infection (PJI), although less common than aseptic loosening, has profound long-term consequences including pain, impaired joint function, reduced quality of life, and even death. PJI can directly cause loosening of the implant and is associated with persistent alterations in bone homeostasis, even after infection eradication, predisposing to subsequent aseptic failure [98–99]. Long-term follow-up demonstrates that patients with PJI have higher rates of revision, lower functional scores, and greater need for assisted living compared to those with noninfected arthroplasties, underscoring the importance of prompt diagnosis and effective management to mitigate adverse outcomes [100].

## 5. Long-Term Clinical Survivorship after Total Hip Arthroplasty

Clinical survivorship between different implants can vary significantly. Surgeons must exercise caution and be aware of the survivorship of each implant when making a selection for a patient, weighing the risks and benefits.

Data from large registries and long-term studies consistently demonstrate that ceramic-on-ceramic (CoC) and highly cross-linked polyethylene (HXLPE)-based metal-on-polyethylene (MoP) bearings achieve the highest implant survival rates at 10 years and beyond. In a Nordic registry analysis of over 158,000 patients, 10-year Kaplan-Meier survival estimates were 95.4% for CoC, 94.3% for ceramic-on-HXLPE, and 94.2% for metal-on-HXLPE, with CoC showing a lower adjusted risk for revision compared to HXLPE-based bearings [101]. Multiple randomized and cohort studies confirm that CoC bearings maintain survival rates above 96% at 10–25 years, with minimal osteolysis and excellent functional outcomes [102–108]. HXLPE-based MoP bearings also demonstrate excellent durability, with wear-related revisions nearly eliminated at 15–20 years [109].

Metal-on-metal (MoM) bearings are associated with significantly lower long-term survivorship and higher revision rates, particularly beyond five years. Registry and meta-analytic data show 10-year survival rates for MoM as low as 85.5%, with increased risk of revision due to adverse local tissue reactions, metallosis, and elevated metal ion levels [110–114]. While small-head MoM designs may perform better in select young, active populations, large-head MoM implants consistently demonstrate inferior outcomes compared to HXLPE and CoC bearings [105]. As a result, MoM use has declined sharply in clinical practice.

Comparative studies with ≥10-year follow-up indicate that CoC and HXLPE-based MoP bearings provide comparable or superior survivorship to conventional MoP and MoM bearings, with lower rates of osteolysis, aseptic loosening, and revision for wear [96–97,106]. Functional outcomes, as measured by Harris Hip Score and WOMAC, are similar across modern bearing surfaces, though CoC may be associated with unique complications such as squeaking and rare ceramic fracture [107–110]. Overall, the long-term evidence supports the use of CoC and HXLPE-based MoP as safe and durable options for most patient populations, with MoM reserved only for specific cases and exceptions.

## 6. Role of Tibial Insert Materials and Design Geometry

In total knee arthroplasty, the durability of tibial insert materials and design geometry is critical. Highly cross-linked polyethylene inserts have reduced wear and osteolysis compared to conventional polyethylene, contributing to lower revision rates for aseptic loosening [111]. Highly cross-linked polyethylene (HXLPE) and compression-molded polyethylene inserts have demonstrated excellent durability, with 14-year survival rates exceeding 98% and no clinically significant osteolysis or insert fracture in large cohorts, including younger and active patients [112]. In general, all-polyethylene tibial components are associated with a significantly lower risk of revision compared to metal-backed modular designs, and this benefit persists across age, sex, and BMI strata. Economic analyses also favor all-polyethylene designs due to lower cost and comparable clinical outcomes [113]. Overall, long-term registry data support the use of HXLPE inserts and modern design geometries for improved survival of the implant.

Furthermore, design geometry, including congruent articular surfaces and optimized constraint, improves load distribution and reduces the risk of mechanical failure. Proper component alignment and patellar tracking are essential to minimize instability, maltracking, and anterior knee pain, which are common causes for surgical revision [114]. Morphometric tibial designs with anatomic bone coverage and squared keel have shown reduced macro-mobility and comparable survivorship to symmetric designs, while ball-in-socket medial conformity with posterior cruciate ligament retention restores native kinematics and maintains baseplate stability [115]. Cementless and hybrid monoblock tibial components, especially those with highly porous surfaces, have achieved 10-year survivorship rates above 96% and minimal aseptic loosening, even in obese and younger patients [116]. Ultracongruent and kinematically aligned inserts also demonstrate high survivorship and excellent functional scores at mid-term follow-up [117].

Another consideration of design geometry involves biomechanical loading and alignment, which are critical indicators of the longevity of tibial inserts. Malalignment, excessive posterior tibial slope, and suboptimal load distribution have been shown to increase contact stresses and risk of loosening, while kinematic alignment and optimal design geometry reduce force imbalances and promote long-term implant survival [118]. Advances in biomaterial science and implant design have led to significant reductions in wear-related revisions, with survivorship exceeding 90% at 10–15 years for contemporary implants [119]. Long-term outcomes of total hip arthroplasty implants are characterized by high survivorship, with meta-analyses of national registry data and case series reporting that approximately 85–90% of hip replacements remain unrevised at 15 years, and about 78% at 20 years [120]. Modern implant designs, including cementless metaphyseal-engaging stems and advanced bearing surfaces, have demonstrated even higher survival rates, with some series reporting over 98% survivorship at 25–28 years in select patient populations [121]. Functional outcomes are consistently favorable, with substantial improvements in pain and activity scores, and patient satisfaction rates exceeding 90% in long-term follow-up [122]. However, patellar tracking and component alignment remain critical technical factors, with malalignment increasing the risk of early failure.

In joint arthroplasty, patellar tracking and component alignment are critical determinants of long-term function and complication rates. In total knee arthroplasty, malalignment—particularly rotational malposition of the femoral or tibial components—can lead to patellar maltracking, increased risk of anterior knee pain, and higher rates of lateral retinacular release or revision [123]. Optimizing femoral component rotation and sagittal alignment has been shown to significantly improve patellar kinematics and reduce patellofemoral complications [124]. Similarly, in hip arthroplasty, precise restoration of the native hip center of rotation and femoral offset is associated with improved patient-reported outcomes and reduced risk of instability or abnormal wear [125].

Overall, the integration of advanced polyethylene materials, anatomic design geometry, and appropriate fixation strategies has led to durable, reliable outcomes in TKA, with revision rates for wear and loosening now being remarkably low in recent clinical practice. The medical literature supports that long-term implant survivorship and functional outcomes in hip and knee arthroplasty are maximized by appropriate implant selection and meticulous attention to component alignment and biomechanics [126]. These factors are especially important in younger, active patients, where the risk of revision and functional demands are higher [127].

## 7. Long-Term Failure Rates after Total Knee Arthroplasty

Long-term mechanical failure rates and revision rates for total knee arthroplasty (TKA) implants are generally low, with 10-year revision rates for modern unconstrained total knee designs reported at 3–6% in large registry studies and meta-analyses [128]. The most common causes of mechanical failure and revision are aseptic loosening (19–30%), instability (7–22%), and polyethylene wear (2–7%), with infection accounting for 15–22% of all revisions; these proportions are consistent across cemented and cementless fixation, as well as across generations of implant design [129].

Regarding specific implants, fixed-bearing TKAs have a lower 15-year major aseptic revision rate (2.7%) compared to mobile-bearing designs (4.1%) [129]. Unicondylar and fully constrained implants have higher lifetime revision risks (13.7–15.4% for patients aged 65–69), while unconstrained TKAs have the lowest risk of lifetime revision (3.6%) [130]. Cemented and cementless TKAs show comparable mid-term revision rates (5.5–5.8% at 9 years), though cementless designs are more often revised for tibial or femoral loosening, and cemented for infection [131]. Modern material and design improvements have reduced polyethylene wear-related revisions but have not significantly changed overall 10-year survivorship compared to older high-performing implants [95–98].

Mechanical failure rates and revision rates increase in younger patients (<55 years), with cumulative revision rates at 15 years reaching 7.8% compared to 1.0% in those ≥75 years [130–132]. This could be presumed to be due to their more active nature and mobility, but additional research is necessary to definitely make this claim. Regardless of age, the tibial component is more frequently revised than the femoral component, and malalignment, instability, and infection remain persistent causes of failure throughout long-term follow-up [74–79].

## 8. Outcomes of Shoulder and Elbow Arthroplasty

For shoulder and elbow arthroplasty, long-term data on hardware survival is more limited compared to the well-established outcomes studied in THA and TKA. With that said, it is important to note that unique biomechanical considerations, such as glenoid component fixation and rotator cuff integrity in total shoulder arthroplasty, and ulnar component stress in elbow arthroplasty, influence implant durability. While modern biomaterials have improved wear resistance, survivorship data beyond 10 years are sparse, and failure mechanisms often differ from those in hip and knee arthroplasty [128]. Further research and registry development are needed to clarify long-term outcomes in the shoulder and elbow.

Specific implants aside, long-term outcomes for shoulder arthroplasty demonstrate high survivorship and sustained functional improvement, particularly with anatomic total shoulder arthroplasty (aTSA) using all-polyethylene cemented glenoid components, which achieves 10-year implant survival rates of 89% and favorable pain and function scores. Stemless and stemmed anatomical shoulder prostheses yield comparable 10-year survival (91.5–95.3%) and functional outcomes, with most revisions related to glenoid complications rather than humeral loosening. Humeral head replacement (HHR) for osteoarthritis provides durable pain relief and improved range of motion at 10–17 years, but is associated with a high rate of glenoid erosion, with some individuals experiencing persistent pain, particularly younger patients and those with eccentric glenoid wear.

Uncemented resurfacing shoulder hemiarthroplasty shows significant functional gains but a high overall revision rate (27%) at 10 years, limiting its use to specific indications only [110,112,115].

Furthermore, biomechanical considerations in shoulder arthroplasty are unique due to the complex interplay of glenohumeral kinematics, rotator cuff integrity, and glenoid component fixation. Glenoid loosening remains the principal mode of failure, particularly in hemiarthroplasty and aTSA, and is exacerbated by rotator cuff dysfunction, eccentric glenoid wear, and suboptimal component positioning. Advances in implant design—such as stemless humeral components, augmented and inlay glenoids, and subscapularis-sparing approaches—aim to preserve bone stock, optimize load transfer, and reduce the risk of loosening and instability, but long-term data remain limited. Radiographic signs of humeral head migration and glenoid medialization are common after a TSA but do not necessarily correlate with inferior clinical outcomes [77–70].

For elbow arthroplasty, total elbow arthroplasty (TEA) provides satisfactory pain relief and functional improvement at ≥10 years, with mean Mayo Elbow Performance Scores in the 80–90 range and 10-year implant survival rates of 75–85% depending on implant type and patient population. Linked TEA designs are preferred for their lower revision rates and greater stability, especially in rheumatoid arthritis and post-traumatic indications, while unlinked designs carry a higher risk of instability. The most common long-term reasons for failure of TES include aseptic loosening, infection, and ulnar nerve complications, with overall revision rates of 10–15% at 10 years. The need to balance stability with range of motion, minimize stress at the bone-implant interface, and accommodate altered loading patterns in the context of soft tissue compromise and bone loss remain significant biomechanical challenges. Early migration of elbow components does not reliably predict long-term failure, and functional outcomes tend to deteriorate over time, especially in younger, more active patients [88–92]. Therefore, additional research is warranted regarding long-term outcomes of hardware in TEA.

In summary, the evolution of biomaterials—particularly highly cross-linked polyethylene and advanced ceramics—has markedly improved the long-term durability and performance of total joint arthroplasty. Selection of bearing surfaces and implant design should be individualized, balancing wear resistance, structural reliability, biomechanical factors, and patient-specific factors to optimize outcomes.

## 9. Long-Term Performance in Spinal Fusion Implants

### 9.1 Spinal Fusion Implant Performance

Spinal fusion implants are critical components in stabilizing the spine and promoting bone fusion following degenerative, traumatic, or deformity-related pathologies. In recent years, advancements in biomaterials have led to the development of various implant types, each with unique mechanical and biological properties. Among the most common are polyetheretherketone (PEEK), titanium, and composite fusion devices, which differ in radiolucency, modulus of elasticity, osseointegration potential, and load-bearing characteristics. Understanding these differences is essential for optimizing implant selection and achieving successful fusion outcomes.

Of the mentioned implant types, polyetheretherketone (PEEK) and titanium are the most widely used interbody cage materials in spinal fusion, each with unique biological and biomechanical properties. PEEK cages are radiolucent and have an elastic modulus closer to cortical bone, which facilitates radiographic assessment of fusion and may reduce stress shielding [132].

However, PEEK is biologically inert and studies have suggested that it demonstrates limited osseointegration, which can compromise long-term fusion integrity [133].

In contrast, titanium cages are radio-opaque and possess a higher elastic modulus, which can increase the risk of stress shielding and subsidence [134]. However, titanium exhibits superior osseointegrative capacity compared to PEEK, promoting direct bone-implant contact and potentially enhancing fusion rates, particularly in lumbar spine fusions [135]. Meta-analyses and randomized trials indicate that titanium cages, particularly those with surface modifications, achieve higher early fusion rates and lower subsidence compared to PEEK in lumbar fusion, while differences in cervical fusion outcomes are less pronounced [136–140].

Composite designs, such as titanium-coated PEEK (TiPEEK), are less common, but nevertheless aim to combine the radiolucency and modulus advantages of PEEK with the osseointegrative properties of titanium. TiPEEK cages have demonstrated superior early bone fusion compared to uncoated PEEK, with similar clinical outcomes at one year [141]. The hybrid construct of composite spinal fusion hardware may facilitate earlier return to activity due to accelerated fusion, though long-term outcomes seem to be similar compared to PEEK and titanium [142].

Radiolucency remains a key advantage of PEEK and composite cages, allowing for more accurate radiographic assessment of fusion and detection of complications [143]. Titanium cages, while radio-opaque, may obscure fusion assessment, often require more advanced imaging methods for accurate evaluation of proper anatomic placement and stability [144].

### 9.2 Subsidence Risk and Suggested Solutions

Subsidence is a potential complication of spinal fusion implants, occurring when the implant sinks into the adjacent vertebral endplates, compromising spinal alignment, reducing fusion rates, and leading to mechanical failure. Subsidence is more likely in cases of poor bone quality or mismatched implant stiffness and is influenced by both implant material and design. PEEK cages have historically demonstrated higher rates of subsidence and revision in the lumbar spine compared to titanium, though solid titanium cages may also be prone to subsidence due to their higher level of stiffness [145]. Regarding subsidence risk, porous and 3D-printed titanium cages are superior alternatives that can mitigate subsidence risk and improve biological stability by reducing modulus and enhancing bone in-growth [146]. Studies have shown that porous titanium cages support both bone on-growth and in-growth, leading to higher fusion rates and lower subsidence at 6–12 months postoperatively [147]. These benefits are attributed to optimized surface architecture and mechanical compatibility with host bone [148]. Overall, these implants are not only optimized to the bone architecture, but help stimulate bone growth and stability that enhance the biomechanical age of the fusion.

Another characteristic of spine implant hardware that can help mitigate subsidence is surface roughness and porosity. Both surface roughness and porosity are critical determinants of bone in-growth and long-term implant stability through their ability to improve osseointegration, load transfer, and long-term implant stability. Increased surface roughness enhances osteoblast attachment, vascularization, and osseointegration, while bulk porosity facilitates bone in-growth and mechanical interlocking [149]. Porous titanium cages, especially those produced via additive manufacturing, demonstrate superior bone in-growth and higher maximum shear stress at the bone-implant interface compared to solid designs [150].

Additional factors that can improve stability include microscale and nanoscale surface modifications, such as nano-etching and microlattice structures. These innovations further promote early fusion and reduce subsidence. Randomized controlled trials have revealed that nano-etched titanium cages achieve significantly higher fusion rates and lower subsidence at 6 months compared to PEEK, with improved segmental stability and decreased pain outcomes [151–155]. These findings highlight the importance of surface topography in facilitating osseointegration and clinical success [156].

Overall, the interplay between surface roughness, porosity, and material composition is central to optimizing interbody cage performance of a spine fusion. Porous 3D-printed titanium and composite cages with engineered surface features provide a biologically favorable environment for bone ingrowth, leading to robust, stable fusions with reduced risk of subsidence and revision surgery [157]. Ongoing advances in biomaterial engineering continue to improve these properties, with the goal of achieving durable, stable spinal fusion.

### 9.3 Spinal Fusion Instrumentation

Instrumentation systems for spinal fusion play a pivotal role in achieving spinal stability, alignment correction, and successful arthrodesis. Modern systems often utilize rod-screw constructs, which provide rigid fixation but are subject to mechanical challenges such as fatigue failure, corrosion, and fretting wear at the implant–implant interfaces. In response to the limitations of rigid fixation, newer technologies have emerged that focus on dynamic stabilization and motion-preserving devices, aiming to maintain physiological spinal motion while reducing stress on adjacent segments. Understanding the biomechanical and material considerations underlying these systems is essential for optimizing implant design, longevity, and patient outcomes.

Rod-screw constructs remain the gold-standard of spinal fusion instrumentation, with titanium alloys, cobalt-chromium, stainless steel, and PEEK rods representing the most commonly used materials. Fatigue life of these constructs is a critical determinant of long-term construct durability. Fatigue life of an implant refers to the amount of loading cycles that the implant can withstand before failure occurs due to material fatigue. In increasing fatigue life, titanium alloys (Ti-6Al-4V) demonstrate superior endurance-limit properties compared to stainless steel, but are sensitive to notch effects and screw-hub design, which may hinder fatigue resistance [158]. Cobalt-chromium rods offer higher stiffness and fatigue strength but may increase stress shielding and adjacent segment degeneration in the long-term [159]. PEEK rods, while less stiff, provide improved load sharing and reduced stress at the rod-screw interface, potentially increasing fatigue life and lowering rates of mechanical failure [160]. This may also provide the added benefit of increased flexibility and functionality.

Corrosion is another significant concern for metallic implants. Long-term retrieval studies show that wear and corrosion are prevalent at rod-screw and connector interfaces, especially in stainless steel constructs, with up to 58% of stainless steel rods exhibiting corrosion, compared to no corrosion observed in titanium rods [161]. Titanium and cobalt-chromium constructs are more resistant to fretting corrosion than stainless steel, with titanium showing the lowest fretting currents and open circuit potential changes under physiological loading [162]. The biological response to corrosion products, including local inflammation and hypersensitivity, remains an area of ongoing investigation [163].

Fretting wear arises from micromotion at rod-screw and connector interfaces, contributing to particulate debris and eventual implant failure. The onset and magnitude of fretting corrosion are material-dependent, with titanium constructs demonstrating superior resistance, followed by cobalt-chromium, and stainless steel being most susceptible [164]. Design factors, such as the elimination of connecting plates and optimization of screw-hub geometry, can reduce stress concentration and maximize fatigue life [165]. While titanium appears to be superior from a corrosion and fretting standpoint, other materials may offer benefit when corrosion is not the most concerning issue regarding spinal fusion stability.

The mechanical performance of rod-screw constructs is also influenced by rod material and design. Rigid rods (stainless steel, titanium) restrict range of motion by a large magnitude (>70% reduction in flexion/extension), while semi-rigid and flexible rods (PEEK, ostaPek) preserve a greater fraction of physiological motion and distribute loads more favorably [166]. However, this increased flexibility may increase the risk of cage subsidence and rod breakage, underscoring the importance of careful patient selection and construct planning at an individual level [167]. Furthermore, clinical studies indicate that screw loosening rates are lower with PEEK rod dynamic fixation compared to titanium rods, with most loosened screws restabilizing over time at the bone interface [168]. The risk of mechanical failure and reoperation is reduced with semi-rigid constructs, though long-term comparative outcomes require further investigation [169].

Some newer implants attempt to preserve motion and reduce pain. Dynamic stabilization and motion-preserving devices, such as Dynesys, DIAM, Isobar TTL, and flexible rod systems, aim to maintain segmental motion and reduce adjacent segment disease. Biomechanical analyses reveal that these devices preserve a portion of physiological range of motion (30–50%), decrease intradiscal pressure, and reduce facet joint forces at instrumented levels [165–170]. Dynamic systems may increase stress at screw insertion zones and apophyses, with device-specific differences in motion restriction and load transmission [171].

Clinical outcomes with dynamic stabilization are variable. Most studies suggest significant improvements in pain and disability scores, with lower rates of adjacent segment degeneration compared to rigid fusion [172]. However, construct failure and reoperation rates remain substantial, and the evidence supporting superior long-term outcomes over rigid fusion is not definitive [173]. The biomechanical utility of pedicle screw-based dynamic devices is limited by their ability to restore load sharing and preserve motion, particularly in cases of severe degenerative disk disease [174]. Finite element and in vivo studies suggest that flexible and dynamic devices may decrease stress on vertebral bodies and adjacent segments; however, this phenomenon may also increase the risk of cage subsidence and device breakage [175]. While allowing for improved flexibility, the limitations in fixation indicate the need for further development of these devices with the goal of demonstrating increased stability in the future.

In summary, the long-term performance of spinal fusion instrumentation systems is governed by material properties, design factors, and biomechanical compatibility with the host bone. Titanium alloys provide the best fatigue and corrosion resistance, whereas PEEK and dynamic devices provide improved load sharing, flexibility, and motion preservation, albeit with increased risk of construct failure. Therefore, the choice of hardware should be individualized, using clinical judgement in balancing mechanical durability, biological response, and patient-centered objectives.

## 10. Spinal Fusion Complications

While spinal fusion remains a mainstay for the management of degenerative disc disease and instability, its long-term success is nonetheless shaped by a spectrum of clinical outcomes and complications. One type of well-recognized sequela of spinal fusion is adjacent segment disease (ASD), a condition such that the spinal segments adjacent to a fused vertebral level develop new degeneration or instability. The development of ASD is multifactorial, involving altered spinal kinematics, increased load transfer across adjacent vertebrae, and patient-specific factors such as pre-existing degeneration and surgical technique utilized [176]. Computational and finite element models have described the mechanism by which fusion increases stress in the annulus fibrosus, nucleus pulposus, and facet joints of adjacent segments, with the proximal segment typically at greatest risk [177]. It is suggested that, since the procedure eliminates motion at the operated segment, the biomechanical stress is transferred to the levels below and above the fusion. Over time, these increased loads may accelerate degeneration of the disks and lead to facet joint arthritis, ligament hypertrophy, or spondylolisthesis at those adjacent vertebral levels. While global and distal spinopelvic alignment parameters do not solely predict ASD, other factors such as preoperative disc health and inclusion of specific levels (e.g., C5-C6 in cervical fusion) may additionally influence risk [170–175].

Indeed, clinical studies consistently demonstrate increased levels of stress and motion at levels adjacent to the fusion, predisposing these segments to accelerated degeneration. Long-term data reveal that radiographic ASD is relatively common, with rates exceeding 25% in some series [172–173]. ASD is associated with adverse patient-reported outcomes, including higher Oswestry Disability Index and lower EQ-5D scores [175]. However, not all radiographic degeneration translates to symptomatic disease, and the relationship between imaging findings, subjective pain and function, and overall clinical impact remains complex [176].

Pseudoarthrosis—the failure of solid bony fusion—remains another significant cause of clinical failure, patient dissatisfaction, and reoperation. Rates of pseudoarthrosis can vary by technique, graft material, and patient factors, and its rates can approach up to 10% or higher in some clinical series. Pseudoarthrosis may present with recurrent discomfort, instability, or hardware failure, but it can also be asymptomatic and only detected on imaging. Diagnosis relies on a combination of clinical assessment and imaging, with CT and dynamic radiographs being the most reliable [177]. The choice of bone graft material influences fusion rates, with local bone showing superior fusion rates compared to autograft, allograft, or synthetic options. Of the latter three options, autograft demonstrates the lowest nonunion risk due to its osteogenic, osteoinductive, and osteoconductive properties, whereas allografts and synthetic substitutes exhibit progressively higher rates of pseudoarthrosis unless supplemented with biologic or cellular enhancers [178].

Another complication is implant migration and failure, defined by the shifting and movement of fusion hardware away from their initial intended placement. While implant migration is less common than the other aforementioned complications, it has clinically significant implications, including nerve impingement, pseudoarthrosis, and nonunion. Implant migration and failure are highly influenced by implant design, material properties, and surgical technique [179]. Systematic reviews have specified that PEEK cages, especially when filled with certain bone substitutes, may be associated with higher rates of subsidence and pseudoarthrosis, while cage loosening and breakage are multifactorial events often related to biological and mechanical factors [150–154]. The need for revision surgery due to implant migration is highest in interbody fusion constructs compared to posterolateral techniques, with no clear advantage in patient-reported outcomes over the long-term [179].

## 11. Diagnosis and Prediction of Complications

Imaging, including radiographs, CT Scan, and MRI, is the standard method in which the long-term assessment of spinal fusion outcomes occurs, including the assessment of fusion status, detection of pseudoarthrosis, and monitoring of adjacent segment changes. In recent years, advanced imaging techniques and computational modeling have allowed for increasingly precise assessments of disc degeneration and tissue remodeling, corroborating accurately with clinical follow-up data [163–168].

Imaging findings on radiographs and CT Scan of ASD include loss of disc height at adjacent segments, osteophyte formation, and facet arthrosis, which progress over time and are influenced by proximity to fusion and preoperative disc health. In addition, anterior or posterior displacement of one vertebra over another will indicate spondylolisthesis. On MRI, decreased signal intensity on T2-weighted images can indicate progressive disk degeneration, while disc degeneration, ligamentum flavum hypertrophy, and facet arthrosis are all suggestive of spinal stenosis. MRI can also detect bone marrow edema in the vertebral body adjacent to the fusion, which may predict early-onset ASD [133,132,138].

Imaging findings that indicate pseudoarthrosis after spinal fusion include absence of bridging bone on CT, persistent motion at the fusion site on dynamic radiographs, lucency around hardware, and increased osteoblastic activity on advanced imaging modalities. CT imaging is the most accurate noninvasive modality for detecting pseudoarthrosis, with findings such as lack of continuous bony bridging across the fusion site, clefts or gaps at the intended fusion level, and possible lucency around implants [172–175].

Regarding implant migration and failure, imaging will usually show clear displacement of interbody cages, rods, or screws from their original postoperative location, sometimes into adjacent anatomical spaces. CT is even more sensitive and is great for detecting more subtle changes in implant migration. Radiolucent halos or zones around screws or cages on CT or radiographs, indicating loss of bone-implant interface stability. SPECT/CT may show increased tracer uptake at sites of loosening. In addition, visible fractures and hardware breakage can be seen clearly and may even suggest underlying pseudoarthrosis [179].

Biomechanical data from long-term follow-up studies reinforce the clinical observations. Fusion restricts motion at the operated segment but increases range of motion and stress at adjacent levels, particularly in the proximal segment. These changes are associated with expansion of moderate-to-high stress areas in the annulus fibrosus and facet joints, providing a mechanistic basis for the development of ASD. Patient-specific modeling and finite element analysis are increasingly used to predict individual risk and guide surgical planning [176].

In all, the long-term performance of spinal fusion implants is shaped by a complex interaction between biomechanical and patient-specific factors. Adjacent segment disease, pseudoarthrosis, and implant migration are the most notable complications, with imaging and biomechanical data providing critical insights into their mechanisms and clinical impact. Robust imaging methods aid with the proper identification of these complications and help guide treatment. Current research into patient-specific modeling, implant design, and biologic augmentation holds promise for lowering complications and improving patient outcomes.

## 12. Future Directions and Emerging Trends

In the future, patterns regarding the long-term performance and resilience of implants and their respective biomaterials for procedures such as total joint arthroplasty and spinal fusion will be heavily influenced by upcoming developments in smart biomaterials and coatings, approaches to regenerative tissue engineering, design guided by artificial intelligence, and obstacles to sustainability and cost effectiveness.

In order to prevent infection and improve hurdles to osseo-integration, smart biomaterials and coating will evolve as they incorporate newer developments in antimicrobial coatings and bioactive interfaces. Silver, bioactive peptides, and general antibiotics as coatings provide a multi-faceted approach to preventing prosthetic joint infections which are a significant obstacle to overcoming implant failure. For an antimicrobial coating to be considered ideal, broad spectrum activity, low bio-toxicity, and provide support in the process of bone healing. In-vivo studies have showcased effectiveness of such coatings with real time clinical application becoming very likely [180]. Implants that are able to elute drugs via the release of antibiotics or growth factors are being meshed into metallic and polymeric apparatuses for local therapy and mitigating any systemic side effects. A multitude of studies have illustrated the viability of incorporating antibiotics into polymeric coatings and metallic implants and are being tested real-time in preclinical models [181]. Nano-structured or composite coatings that can account for a bioactive interface are being synthesized to match the biological characteristics of native tissues, modify immune responses, and assist in the process of osteogenesis. The goal of these coatings is to improve integration of implants and their respective longevity via enhancement of bone-to-implant interactions. While research within this field is an ongoing process, many products have portrayed promising results in pre-clinical trials [182].

Engineering of tissues with regenerative properties are poised to gain significant prominence with the integration of stem cell-based therapies. Scaffolds that integrate mesenchymal stem cells (MSCs) or other growth factors are theorized to amplify bone regeneration and advance the healing process not only in joint arthroplasty, but also in applications to spinal fusions [183]. Biodegradable and porous scaffolds, designed via additive techniques, are utilized as a medium for cell and bioactive molecule delivery to the desired surgical site. Such scaffolds provide a foundation for scaffold-directed bone regeneration and will eventually be resorbed systemically as concurrent native tissue is formed [184]. Crossbreeds that integrate both inorganic and organic phases, such as hydroxyapatite and polymers, are in the process of being developed for optimization of mechanical strength and biological agreement. Ultimately, the end-goal is to synthesize a synthetic biomaterial that can mimic living characteristics with adaptability to the host’s domain [185].

Artificial intelligence (AI)-lead design and stimulatory environments will play an increasing role in biomaterial implant design and development. Computational programs such as finite element modeling (FEM) are already used as a predictor of mechanical performance, evaluating stress distribution within the surrounding bone-implant space. In order to evaluate for potential failure scenarios via wear or stress shielding, FEM can be used which allows for design optimization before real-time manufacturing trials [186]. Clinical outcomes, imaging, and material testing results within large datasets can be run through various machine learning algorithms to better predict various models of implant performance. This approach identifies an optimal device for a given patient’s characteristics which in turn mitigates future complications or, in some cases, complete implant failure [187]. Development of high-throughput screening platforms, such as automated cell culture systems and micro-fluidic devices are key to a rapid approach to screening new biomaterials and regenerative therapies. These techniques will streamline discovery of usable biomaterials and curtail time and cost associated with bringing these products to real-time markets [188].

Viability and cost-efficiency are pivotal considerations regarding the future of biomaterials and implant design. Analysis of a biomaterial implant’s life cycle will be incorporated into considerations of environmental impact via use and disposal, with an emphasis on curtailing waste and energy consumption [189]. Such developments are already in the design phase, with biodegradable spinal fusion cages being manufactured and tested to address the problems of foreign bodies within the spine. Implants that are readily absorbable by human tissue will reduce the need for revision surgeries and improve overall post-procedure outcomes [188]. In terms of cost-effectiveness, surface modification and increasingly streamlined manufacturing techniques are being considered to improve patient outcomes without increasing material and healthcare costs [180]. In order to address larger trends in sustainable healthcare, efforts to improve the environmental footprint and the recyclability of biomaterial implants will need to be addressed, with emphasis on reducing the use of critical raw materials.

## 13. Critical Analysis and Synthesis

Long-term outcomes across biomaterials and implant designs have drastically improved over the course of time due to innovations in material science, surface modification, and biomechanics. Currently, titanium alloys, ceramics, and highly crosslinked polyethylene are offering the best balance for both durability and stability, however, for the future, composite and 3D-printed biomaterials that are more tailored to the patient are showing extended durability and minimized complications [190].

The current gold standard for orthopedic and spinal implants is the titanium/titanium alloys due to their biocompatibility, mechanical strength, and corrosion resistance. Due to these imperative properties, it has been proven to increase survival rates to 90–95% at the duration of 10–15 years [191]. Some limitations to this implant is that it has the tendency to release trace ions that could potentially be cytotoxic as well as stress shielding which is defined as a reduction of physiological stress on the surrounding bone which can lead to further bone loss and deterioration [192].

Cobalt-chromium implants are utilized for their high wear resistance and exhibit fatigue properties, mainly for hip and knee arthroplasties. This biomaterial has shown a 20 year survival rate of 85–90% [193]. One benefit of using this material is that due to minimal mechanical wear, there is less component release. Some limitations, though minimal, are ion release which can lead to local toxicity, and if worsened, systemic toxicity [194].

Stainless steel, though cost effective, is being largely phased out for more permanent options due to the limitations of it being far inferior due to high poor corrosion resistance. The leeching of nickel and chromium ion release from the implant led to higher revisions and faliure rates [195].

UHMWPE and HXLPE have improved over the past decades. UHMWPE was known to cause particle induced osteolysis which was causing an increase in surgical revisions, until the advent of the HXLPE which made substantial improvements in order to improve the wear resistance, creating a 90% success rate and lasting up to 15–20 years [196]. A concerning limitation of oxidative degradation but it was discovered that using vitamin E antioxidants can stabilize the free radicals and improve durability [197].

PEEK is known for its excellent load sharing and reduced stress shielding. The bone-like structure promotes physiological load transfer and minimizes degeneration [198]. Limitations for this method is that the inert surface hinders osteo-integration which increases risk of improper fusion. This has led to improvements in surface modifications that can improve long term fusion stability and reduced revision rates [199].

Ceramics have been shown to exhibit the most superior wear resistance and the most impressive biocompatibility, with minimal particle generation. These materials have been shown to have survival rates higher than 95% at 15–20 years without need for surgical revision [200]. Limitations to these materials are that they are brittle and have a high fracture rate, but due to their application, they are sought out in young patients due to their longevity [201].

As for implantation design, HA and bioactive coatings are utilized to enhance the early growth of bone and long-term fixation by promoting osteoconduction. These coatings have been shown to improve the osteo-integration and lower septic rates with titanium and cobalt-chromium implants [202]. A limiting factor for these coatings is their durability which delaminate over time which can reduce structural integrity. This has been an ongoing issue as further research is being conducted to determine the effectiveness of materials in order to improve their stability and increase longevity of outcomes [203].

Though these materials are very effective for utilization in modern healthcare, one of the largest gaps in current knowledge is limited by the lack of prolonged data dealing with the long-term effects and comparative data [204]. The majority of these technological advancements have been recent revelations that have not yielded prolonged results that still have yet to be fully understood [205]. Narrowing the variation of the long-term results and effects of these materials and coatings is an important future direction for further research. Another limitation of these variable materials is that the mechanobiological process varies drastically which makes it difficult to quantify the bone growth in order to determine the outcome potential of these repairs [17]. Due to the nature of the repairs and different variable factors such as bone quality, metabolic health, and local vascularity also make it increasingly difficult to monitor growth and longevity of implants. Finally, another limitation to address is the insufficient evidence that links the implants material properties to the meaningful quality of life improvements that come from the procedures [18]. There are minimal studies and scientific data that report on the biomaterial characteristics in relation to functional recovery, pain reduction, and improving patient outcomes which makes it difficult to determine how these confounding factors interact with the implants and affect the long-term outcome.

Finally, a discussion on methodological heterogeneity in clinical and in-vitro studies is also another limiting factor due to reliability issues, compatibility issues, and lack of translational evidence of value. Primarily, the study design widely varies in design, sample size, and follow up duration which makes it difficult to create comparisons between the materials and implantations [206]. In addition, different studies tend to use different outcome metrics due to narrow scopes/definitions of success, which can lead to results that are not representative. Another issue with different study designs is the variability of patient selection which introduces many different confounding factors. For example, younger patients will have better outcomes compared to patients that are older, obese, or have other comorbidities. This becomes an issue when these studies end up generalizing the results, which can lead to skewed results [207]. Lastly, in vitro studies that are used to evaluate these materials are marked by heterogeneity in testing protocols and stimulating a physiological environment. This method is used for corrosion testing, mechanical testing, and osseointegration studies to study the result in a live environment. The discrepancies that occur make it challenging to extrapolate these preclinical findings and relate to clinical outcomes due to all of the compounding factors that are in play. There’s still a lot of inconsistency in how studies on implant materials are designed and reported, which makes it hard to compare results or draw clear conclusions about which options work best [208]. Differences in patient populations, testing methods, and definitions of success often lead to mixed or incomplete findings. In order to improve this, research needs to be more standardized—using clear, consistent criteria for outcomes, testing under similar conditions, accounting for patient and implant differences, and being transparent about material details [209,210]. Larger, long-term, and independent studies will also be key to getting more reliable and comparable results.

## 14. Conclusions

The longevity of orthopedic and spinal implants is influenced by the integration of a multitude of advanced biomaterials, implant design, and biomechanical factors tailored to each patient. New materials including titanium alloys, ceramics, and highly cross-linked polyethylene lead to the improvement of durability, osseointegration, and wear resistance. Surface modifications such as 3D-printed constructs and porous architectures improve mechanical stability and fusion rate success. Although there are significant advancements in the field, complications including aseptic loosening, wear-particle osteolysis, pseudoarthrosis, adjacent segment disease, and infection continue to remain major contributors to implant failure. Further emphasizing the need for patient-specific implant selection based on factors such as age, activity level, and bone quality. Future progress will rely on smart antimicrobial and bioactive coatings, regenerative scaffold technologies, and AI-driven design optimization. Further standardized clinical studies capable of linking biomaterial properties to positive patient outcomes are still needed.

## Key Points

- This is a critical review of the historical progression of orthopedic biomaterials and their impact on durability and longevity.
- Latest information is presented on the mechanical and biologic requirements of successful orthopedic implants such as load distribution and fatigue resistance.
- A comparative analysis is provided on the most frequently used biomaterials including titanium alloys, stainless steel, and cobalt chromium to assess advantages and limitations with each material.
  - A critical assessment is presented on the effect of polyethylene implants and advancements in highly cross-linked and antioxidant stabilized UHMWPE to reduce wear and oxidative stress
  - The implant design principles such as modular versus monoblock constructs, cemented versus non cemented, and optimization of geometric considerations are critical to reduce stress shielding.
  - Surface engineering techniques including porous coatings, hydroxyapatite application, and bioactive modifications in the role of osseointegration are presented.
  - The complications with implant materials which include aseptic loosening, osteolysis, corrosion, and periprosthetic fractures are explored.
  - Overall, the article highlights new innovations in the field of orthopedic implants such as additive manufacturing, patient-specific implants, and biomimetic materials to improve long-term survivorship and improve functionality.
